# Clinical Validation of a Handheld Deep Learning Tool for Identification of Glaucoma Medications

**DOI:** 10.18502/jovr.v19i2.13983

**Published:** 2024-06-21

**Authors:** Christopher D. Yang, Jasmine Wang, Ludovico Verniani, Melika Ghalehei, Lauren E. Chen, Ken Y. Lin

**Affiliations:** ^1^University of California, Irvine School of Medicine, Irvine, CA, USA; ^2^Gavin Herbert Eye Institute, Department of Ophthalmology, UC Irvine, CA, USA; ^3^Department of Computer Science, UC Irvine, CA, USA; ^4^Department of Biomedical Engineering, UC Irvine, CA, USA; ^6^Christopher D. Yang: https://orcid.org/0000-0002-2090-1880; ^7^Ken Y. Lin: https://orcid.org/0000-0002-6467-7219; ^8^These two authors contributed equally to this work.

**Keywords:** Convolutional Neural Network, Deep Learning, Glaucoma, Medication Tools

## Abstract

**Purpose:**

To validate a convolutional neural network (CNN)-based smartphone application for the identification of glaucoma eye drop medications in patients with normal and impaired vision.

**Methods:**

Sixty-eight patients with visual acuity (VA) of 20/70 or worse in at least one eye who presented to an academic glaucoma clinic from January 2021 through August 2022 were included. Non-English-speaking patients were excluded. Enrolled subjects participated in an activity in which they identified a predetermined and preordered set of six topical glaucoma medications, first without the CNN and then with the CNN for a total of six sequential measurements per subject. Responses to a standardized survey were collected during and after the activity. Primary quantitative outcomes were medication identification accuracy and time. Primary qualitative outcomes were subjective ratings of ease of smartphone application use.

**Results:**

Topical glaucoma medication identification accuracy (OR = 12.005, *P* < 0.001) and time (OR = 0.007, *P* < 0.001) both independently improved with CNN use. CNN use significantly improved medication accuracy in patients with glaucoma (OR = 4.771, *P *= 0.036) or VA ≤ 20/70 in at least one eye (OR = 4.463, *P *= 0.013) and medication identification time in patients with glaucoma (OR = 0.065, *P *= 0.017). CNN use had a significant positive association with subject-reported ease of medication identification (X^2^(1) = 66.117, *P* < 0.001).

**Conclusion:**

Our CNN-based smartphone application is efficacious at improving glaucoma eye drop identification accuracy and time. This tool can be used in the outpatient setting to avert preventable vision loss by improving medication adherence in patients with glaucoma.

##  INTRODUCTION

The outpatient management of glaucoma relies heavily on lowering intraocular pressure (IOP), a major modifiable risk factor, through pharmacologic and procedural interventions.^[1]^ A mainstay of glaucoma management is the administration of topical medications (eye drops) that act on different physiologic pathways to reduce IOP.^[2]^ However, the identification of these medication bottles is challenging for patients with glaucoma and pronounced vision loss.^[3]^

Patients with glaucoma and poor visual acuity (VA) or those suffering from cognitive decline may find it difficult to identify eye drops, leading to medication noncompliance and inadequate treatment. Glaucoma eye drop labels wear off over time and medication bottles share similar shapes and sizes, making it difficult for patients with vision loss to distinguish between them based on physical characteristics. The fine print on topical ophthalmic medications can also be difficult for patients with poor vision to read and can interfere with accurate medication identification.^[4]^ Reliance on bottle cap color also yields inconsistent identification. Several studies have shown that among patients with glaucoma who find bottle cap color necessary to differentiate between their medications, not all of them are able to do so accurately.^[3,5,6]^

All these factors increase the risk of medication noncompliance in patients with glaucomatous vision loss, which can impair quality of life or increase the risk of ophthalmic comorbidities.^[7]^ Several studies have shown that medication noncompliance has a positive association with glaucomatous visual field progression.^[8,9]^ The California Department of Motor Vehicles mandates that all individuals applying for a driver's license must meet a VA screening threshold of 20/40 in one eye and at least 20/70 in the other eye.^[10]^ Medication noncompliance in patients with glaucoma can prevent access to driving, a crucial mode of transportation that, when absent, can negatively affect patients' independence and overall mobility. This suggests that interventions to improve medication compliance in patients with glaucoma are critical for improving quality of life.

Convolutional neural networks (CNNs) are a class of deep learning neural networks commonly applied to image analysis and offer a novel approach to addressing medication noncompliance in patients with diabetes and glaucoma.^[11]^ With training and optimization, CNNs can accurately classify medications and differentiate among them.^[12]^ We previously developed a smartphone application that integrates a MobileNet V2 CNN trained on images of glaucoma eye drops bottles that can identify them with high sensitivity and specificity.^[13]^

The present study aims to improve our previously described CNN model and introduce a streamlined and user-friendly iOS mobile application to the clinical setting for validation and patient feedback. We utilize an updated model that integrates text recognition capabilities and incorporate patient demographic factors and relevant ophthalmic history to explore the efficacy of our CNN as it relates to pre-existing comorbidities. We also employ qualitative surveys to evaluate patient perceptions of application ease of use via standardized participant questionnaire.

##  METHODS

### Study Design

A standardized medication identification activity was performed by patients eligible for our study at an academic outpatient glaucoma clinic from October 2021 through August 2022. Our study was approved by the institutional review board of the University of California, Irvine. Medical data collected included patient demographics such as age, sex, and pre-existing conditions such as glaucoma diagnosis and severity, VA, and prior glaucoma eye drop use. Primary outcomes of interest included eye drop bottle identification accuracy and time, as well as patient-reported ratings of ease of smartphone application use. All tenets of the Declaration of Helsinki were followed. Data collection and evaluation were conducted in accordance with the Health Insurance Portability and Accountability Act.

### Subject Selection

All patients who verbally consented to study enrollment were eligible for inclusion, regardless of VA or glaucoma stage. Adult patients with poor vision were categorized on the basis of VA of 20/70 or worse in one eye as recorded in the electronic medical record from their last clinic visit just prior to enrollment and participation in the study, as per the driver's license vision screening standard established by the California Department of Motor Vehicles.^[10]^ Exclusion criteria included patients who did not speak English. Of the 81 patients screened for study eligibility, 68 met criteria for inclusion.

### Informed Consent

Study participants verbally consented in a private clinic room at the point of care. Institutional Review Board (IRB) and Ethics Committee approval were obtained under UC Irvine IRB#20216783.

### Convolutional Neural Network Training

A total of 9860 mobile-captured images were taken to create the training dataset for our CNN model. These images included nine commonly-prescribed eye drop medications: Pred Forte, Vigamox, Rocklatan, Rhopressa, Latanoprost, Dorzolamide, Cosopt, Combigan, and Alphagan. These medications were selected based on ready availability and use in our eye clinic. Images were captured with bottles in various backgrounds, distances, lighting conditions (natural and artificial), bottle orientations, and image resolutions. Eye drop bottles were rotated throughout the images such that approximately half of them did not contain the medication name in frame. A second dataset of 1088 images was created for model validation using the same parameters as the first dataset. The CNN architecture MobileNet V2 was found in our prior study to have the highest image prediction accuracy and shortest image processing time when compared against six other modern architectures.^[13]^ In the present study, we utilized an updated version (MobileNet V3), which was finely tuned by unfreezing the last four layers of the model and adding a dropout layer for maximum optimization. Other hyperparameters such as learning rate, epoch magnitude, and weight decay were also calibrated. In addition, a text recognition system was built on top of Google's computer vision API and included smart word matching from dictionaries with unique tokens from each bottle label [Supplementary Table 1].

### Smartphone Application Design

The CNN model was integrated with a custom interpolation algorithm that combines model output and text processing results from previous results to provide greater image prediction accuracy and subsequently compressed into a Keras HDF5 file and converted into a format compatible with Apple's core machine learning infrastructure to run natively on iOS devices. The trained model was then embedded into a simple user interface coded in Swift using UI Kit, a collection of user interface components. This yielded an iOS smartphone application that, when opened by a mobile device user, immediately begins capturing and processing 24 image frames per second. The output from the iOS application includes written text in large font and audio of the eye drop medication name.

### Standardized Medication Identification Activity

Study participants were asked to first identify a set of three branded glaucoma eye drop medications (Alphagan, Dorzolamide, Latanoprost) without using the CNN smartphone application. Immediately afterward, they were then asked to identify a different set of three branded glaucoma eye drop medications (Combigan, Pred Forte, Rhopressa) while using the CNN smartphone application. Patients were not refracted and near vision was not specifically tested in this study. All images were captured using an iPhone 11 Pro (Apple Inc, Cupertino, CA) at maximum allowable screen brightness and volume. Eye drop bottles were presented to each participant in the same temporal sequence. Time to identification (defined as the time between placement of the eye drop bottle on the clinic table and participant verbalization of the medication name) and identification accuracy were recorded as primary study endpoints. Because each participant took part in the medication identification activity with and without the CNN smartphone application, they acted as their own controls. Therefore, each participant yielded a total of six measurements, yielding a high-powered crossover, within-subjects study design.

### Patient Questionnaire

Study participants were asked to rate the ease of identifying the eye drop bottles and one open-ended survey question following completion of the first half of the eye drop bottle identification activity without the smartphone application. After completion of the second half of the eye drop bottle identification activity with the smartphone application, subjects were again asked to rate the ease of identifying the eye drop bottles. All survey questions interrogating subjective participant ratings of ease used an ordinal 1-5 scale, with 1 being very easy and 5 being very difficult [Supplementary Tables 2 & 3].

### Chart Review

Participant demographic variables and ophthalmic history were determined via filtered search of the UC Irvine electronic medical record. Glaucoma severity was defined using International Statistical Classification of Diseases and Related Health Problems, Tenth Revision (ICD-10) codes. Prior medication user status was self-reported and defined according to prior use of any of the glaucoma eye drop medications used in the Standardized Medication Identification Activity (Alphagan, Dorzolamide, Latanoprost, Combigan, Pred Forte, or Rhopressa). Chart reviewers were trained to collect data points, which included demographic variables and VA, from the EMR and utilized a standardized data collection form developed *a priori*. After preliminary data collection, chart reviewers removed erroneous data or missing data points.

### Outcome Measures

Primary quantitative outcomes were medication identification accuracy and time. Primary qualitative outcomes were subjective ratings of ease of smartphone application use collected from participant questionnaires.

### Statistical Analysis

Data were analyzed using the SPSS 28.0 (IBM Corp; Armonk, NY). Descriptive statistics were run to evaluate the baseline characteristics of study participants. Within-subjects binary logistic regression and linear regression were run to analyze various demographic and clinical characteristics to determine the significance of their relationships to medication identification accuracy and time, respectively. Descriptive statistics and Chi-squared tests were used in the analysis of participant questionnaire responses. Associations were reported as odds ratios.

##  RESULTS

Several tests were conducted with images from our image validation dataset to compare the image prediction accuracy and average processing time of the CNN model alone, the text recognition model alone, and the combined text recognition and CNN model. The text recognition system alone was able to find text 75.92% of the time, whereas the CNN model alone had a prediction accuracy of 96.14%. The combined model had a prediction accuracy of 99.07% [Table 1]. Furthermore, the average processing time was 0.248 s for the text recognition system, 0.103 s for the CNN model, and 0.213 s for the combined model. The absence of an increase in average processing time for the combined model compared to the text recognition system alone is likely due to greater computational power associated with a combined CNN architecture. Because the combined CNN and text recognition system exhibited superior prediction accuracy and negligible processing time differences, it was ultimately utilized for the iOS application.

**Table 1:** CNN model accuracy and time tests


	**Processing accuracy**	**Processing time (s)**
Text recognition alone	75.92%	0.248
CNN model alone	96.14%	0.103
Combined model	99.07%	0.213
	
	
CNN, convolutional neural network; S, seconds		

**Figure 1:**
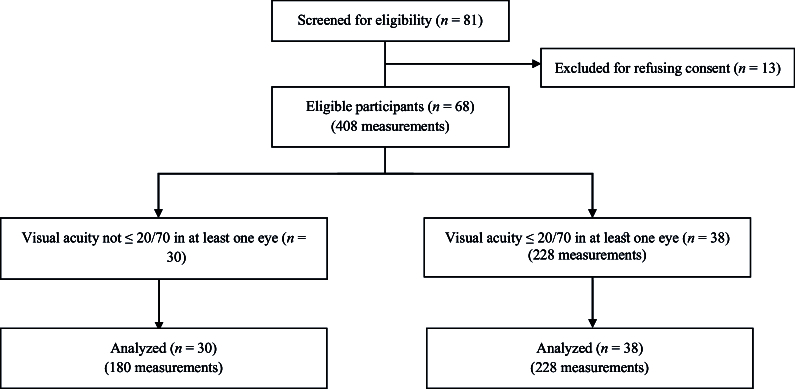
Inclusion criteria flow chart.

Of the 81 patients screened for eligibility for this validation study, 13 were excluded because they did not consent to enrollment. Of the 68 remaining eligible patients, 30 (44.1%) did not have a VA of ≤ 20/70 in at least one eye at the time of presentation, while 38 (55.9%) did. Standardized glaucoma eye drop medication identification activities and patient questionnaires were administered to all 68 study participants [Figure 1].

The average age of study participants was 70.87 years. A majority of study participants were male (67.6%), had a pre-existing glaucoma diagnosis (85.3%), and demonstrated a VA of 20/70 or worse in at least one eye during their clinic visit (55.9%). Moreover, 20.6% of study participants had glaucoma classified as mild, 11.7% as moderate, 27.9% as severe, and 25% as indeterminate. A majority of study participants reported being prior users of medications utilized in the Standardized Medication Identification Activity (76.5%) [Table 2].

**Table 2:** Baseline demographic and clinical characteristics


	**Descriptive statistics (** ***n*** ** = 68)**
Age (yr) Mean Standard deviation	70.87 14.54
Sex Male Female	46 (67.6%) 22 (32.4%)
Diagnosed glaucoma? No Yes	10 (14.7%) 58 (85.3%)
Glaucoma severity None At risk Mild Moderate Severe Indeterminate	5 (7.4%) 5 (7.4%) 14 (20.6%) 8 (11.7%) 19 (27.9%) 17 (25%)
VA in at least one eye ≤ 20/70 > 20/70 History of prior topical glaucoma medication use No Yes	38 (55.9%) 30 (44.1%) 16 (23.5%) 52 (76.5%)
	
	
white<bcol>2</ecol>yr, years; n, number; VA, visual acuity

Within-subjects univariate and multivariate binary logistic regression analyses of eye drop identification accuracy using prior glaucoma diagnosis, prior eye glaucoma eye drop use, VA ≤ 20/70 in at least one eye, and CNN smartphone application use as input factors is presented in Table 3. VA ≤ 20/70 in at least one eye was found to be an independent predictor of decreased medication identification accuracy (OR = 0.330, *P* < 0.001), while CNN smartphone application use was found to be an independent predictor of increased medication identification accuracy (OR = 12.005, *P* < 0.001). The multivariate model was significant overall (X^2^(1) = 13.454, *P* < 0.001), with VA ≤ 20/70 in at least one eye as a significant predictor of decreased medication identification accuracy (OR = 0.213, *P* < 0.001) and CNN smartphone application use as a significant predictor of increased medication identification accuracy (OR = 2E11, *P* < 0.001). Prior glaucoma diagnosis was associated with decreased medication identification accuracy, but this finding was not significant (OR = 0.391, *P *= 0.151). Prior glaucoma eye drop use was associated with increased medication identification accuracy, but this finding was also not significant (OR = 1.772, *P *= 0.130).

**Table 3:** Binary logistic regression analysis of patient factors in relation to eye drop bottle identification accuracy


**Variable of interest**	**Univariate analysis**			**Multivariate analysis**	
	**OR (95% CI)**	** ${\;}^{2}$ value (DOF)**	***P*** **-value**	**OR (95% CI)**	** ${\;}^{2}$ value (DOF)**	***P*** **-value**
Intercept	–	–	–	11.827 (3.159–44.276)	13.454 (1)	< 0.001***
Prior glaucoma diagnosis	0.411 (0.133–1.276)	2.366 (1)	0.124	0.391 (0.108–1.410)	2.059(1)	0.151
Prior glaucoma eye drop use	0.830 (0.436–1.542)	0.380 (1)	0.538	1.772 (0.845–3.716)	2.292 (1)	0.130
VA ≤ 20/70 in at least one eye	0.330 (0.168–0.648)	10.388 (1)	< 0.001***	0.213 (0.086–0.525)	11.301 (1)	< 0.001***
CNN smartphone application use	12.005 (4.424–32.578)	23.809 (1)	< 0.001***	2E11 (3.498E10–1.144E12)	855.454 (1)	< 0.001***
CNN smartphone application use and prior glaucoma diagnosis	–	–	–	4.771 (1.108–20.551)	4.399 (1)	0.036*
CNN smartphone application use and VA ≤ 20/70 in at least one eye	–	–	–	4.643 (1.386–15.558)	6.193 (1)	0.013*
	
	
CI, confidence interval; DOF, degrees of freedom; OR, odds ratio *P < 0.05; ***P < 0.001						

**Table 4:** Linear regression analysis of patient factors in relation to eye drop bottle identification time


	**Univariate analysis**			**Multivariate analysis**	
**Variable of interest**	**OR (95% CI)**	** ${\;}^{2}$ value (DOF)**	***P*** **-value**	**OR (95% CI)**	** ${\;}^{2}$ value (DOF)**	***P*** **-value**
Intercept	–	–	–	671.060 (83.840–5371.234)	37.618 (1)	< 0.001***
Prior glaucoma diagnosis	49.760(11.482–215.658)	27.270 (1)	< 0.001***	198.180 (19.214–2044.095)	19.735 (1)	< 0.001***
Prior glaucoma eye drop use	2.703 (0.419–17.418)	1.094 (1)	0.296	0.466 (0.081–2.672)	0.733 (1)	0.392
VA ≤ 20/70 in at least one eye	3.876 (0.527–28.505)	1.771 (1)	0.183	4.323 (0.236–79.199)	0.974 (1)	0.324
CNN smartphone application use	0.007 (0.002–0.030)	46.308 (1)	< 0.001***	0.080 (0.011–0.609)	5.956 (1)	0.015*
CNN smartphone application use and prior glaucoma diagnosis	–	–	–	0.065 (0.007–0.615)	5.674 (1)	0.017*
CNN smartphone application use and VA ≤ 20/70 in at least one eye	–	–	–	0.716 (0.048–10.777)	0.058 (1)	0.809
	
	
CI, confidence interval; DOF, degrees of freedom; OR, odds ratio *P < 0.05; ***P < 0.001						

**Table 5:** Descriptive statistics of questionnaire ease of use responses


	**Without CNN smartphone application**	**With CNN smartphone application**	
**Survey responses (** ***n*** ** = 68)**	**Ease of identifying the eye drop bottle with your naked eye?**	**Ease of identifying the eye drop bottle using the CNN smartphone application?** * *	
Very easy (1)	12 (17.1%)	52 (74.3%)	
Easy (2)	13 (18.6%)	11 (15.7%)	
Okay (3)	20 (28.6%)	5 (7.1%)	66.117 (8)	< 0.001***
Difficult (4)	11 (15.7%)	0 (0%)	
Very difficult (5)	12 (17.1%)	0 (0%)	
	
	
CNN, convolutional neural network; DOF, degrees of freedom ***P < 0.001				

**Table 6:** Perceived medication identification difficulty predicts medication identification accuracy and time


**Survey responses**	**OR (95% CI)**	** ${\;}^{2}$ value (DOF)**	***P*** **-value** * *	**OR (95% CI)** * *	${\;}^{2}$ **value (DOF)**	***P*** **-value**
Ease of medication identification *without* CNN smartphone application (1 = very easy; 5 = very difficult)	0.539 (0.440–0.659)	36.206 (1)	< 0.001***	2.291 (1.329–3.948)	8.914 (1)	0.003**
Ease of medication identification *with* CNN smartphone application (1 = very easy; 5 = very difficult)	0.628 (0.455–0.866)	8.051 (1)	0.005**	8.224 (1.520–44.483)	5.985 (1)	0.014*
	
	
CI, confidence interval; CNN, convolutional neural network; DOF, degrees of freedom *P < 0.05; **P < 0.01; ***P < 0.001						

CNN smartphone application use in patients with glaucoma was associated with a significant increase in medication identification accuracy compared to patients with glaucoma who did not use the CNN smartphone application (OR = 4.771, *P *= 0.036). CNN smartphone application use in patients with VA ≤ 20/70 in at least one eye was associated with a significant increase in medication identification accuracy compared to patients with VA ≤ 20/70 in at least one eye who did not use the CNN smartphone application (OR = 4.463, *P *= 0.013).

Within-subjects univariate and multivariate linear regression analyses of eye drop identification time using prior glaucoma diagnosis, prior glaucoma eye drop use, VA ≤ 20/70 in at least one eye, and CNN smartphone application use as input factors is presented in Table 4. Prior glaucoma diagnosis was found to be an independent predictor of increased medication identification time (OR = 49.760, *P* < 0.001), while CNN smartphone application use was found to be an independent predictor of decreased medication identification time (OR = 0.007, *P* < 0.001). The multivariate model was significant overall (X^2^(1) = 37.618, *P* < 0.001). Prior glaucoma diagnosis was associated with a significant increase in medication identification time (OR = 198.810, *P* < 0.001). In contrast, CNN smartphone application use was associated with a significant decrease in medication identification time (OR = 0.080 *P *= 0.015). Prior glaucoma eye drop use was associated with a decrease in medication identification time, but this finding was not significant (OR = 0.466, *P *= 0.392). VA ≤ 20/70 in at least one eye was associated with an increase in medication identification time, but this finding was not significant (OR = 4.323, *P *= 0.324).

CNN smartphone application use in patients with glaucoma was associated with a significant decrease in medication identification time compared to patients with glaucoma who did not use the CNN smartphone application (OR = 0.065, *P *= 0.017). CNN smartphone application use in patients with VA ≤ 20/70 in at least one eye was associated with a decrease in medication identification time compared to patients with VA ≤ 20/70 in at least one eye who did not use the CNN smartphone application, but this finding was not significant (OR = 0.716, *P *= 0.809).

Chi-square analysis of patient responses to a validated questionnaire developed to assess self-reported ease of eye drop identification revealed a significant improvement in ease with the CNN smartphone application compared to without (X^2^(1) = 66.117, *P* < 0.001). Furthermore, 35.7% of patients identifying eye drops without the CNN smartphone application reported the identification process as easy or very easy, and this value increased to 90% in patients identifying eye drops with the CNN smartphone application [Table 5].

A separate Chi-square analysis was run to evaluate the relationship between patient questionnaire responses and eye drop identification accuracy and time [Table 6]. Self-reported ratings of difficulty with (OR = 0.628, *P* < 0.001) and without (OR = 0.539, *P* < 0.001) the CNN smartphone application negatively correlated with medication identification accuracy, although this effect was more pronounced in patients not using the CNN smartphone application. In contrast, self-reported ratings of difficulty with (OR = 8.224, *P *= 0.003) and without (OR = 2.291, *P *= 0.014) the CNN smartphone application positively correlated with medication identification time; this effect was more pronounced in patients using the CNN smartphone application.

##  DISCUSSION

In this validation study, we systematically evaluated a handheld smartphone-based CNN capable of identifying prescription glaucoma eye drop medications by combining real-time image input with a text recognition model in an outpatient clinical setting. We observed that medication identification accuracy (OR = 12.005, *P* < 0.001) and time (OR = 0.007, *P* < 0.001) both independently improve with use of this smartphone-based CNN, which suggests this tool has the potential to improve medication compliance in patients who utilize topical ophthalmic medications. To the best of our knowledge, this is the first clinical report of a handheld CNN that can be used at the point-of-care to help patients identify glaucoma eye drop bottles.

Prior studies have described medication noncompliance as a serious issue that can contribute to irreversible vision loss in patients with moderate-to-severe glaucoma.^[14,15]^ These patients are often prescribed multiple topical medications and, because of the visual nature of their disease, many find it difficult to distinguish between them. Other factors that may preclude consistent adherence to glaucoma eye drop administration include low self-efficacy and motivation, poor memory, aging, and complicated schedules of medication administration.^[16,17]^

In our previous work, we described the development of a smartphone-based CNN and characterized the training of MobileNet V2 to quickly and accurately identify six different glaucoma eye drop medications based on visual input.^[13]^ Since the publication of this work, we have created a new model that integrates a robust text recognition system with an updated CNN model to recognize ophthalmic medication bottles, which improved the image prediction accuracy of our mobile application from 86.2% to 99.07% and decreased the image processing time from 3.45 s to 0.213 s per image.

In the present validation study, our multivariate analysis found that this CNN significantly improves medication accuracy in patients with glaucoma (OR = 4.771, *P *= 0.036) or VA ≤ 20/70 in at least one eye (OR = 4.463, *P *= 0.013) and medication identification time in patients with glaucoma (OR = 0.065, *P *= 0.017). Study participants described the CNN as making medication identification easier (X^2^(1) = 66.117, *P* < 0.001), suggesting that real-world distribution of this tool in the outpatient setting could potentially avert preventable vision loss and morbidity in patients with glaucoma or poor vision.

Although medication noncompliance is a known issue among glaucoma specialists, there have been few attempts to address this problem outside of patient education initiatives. One study found that telemedicine-based patient reminders marginally improved medication adherence in patients with glaucoma.^[18]^ Another study found a significant improvement in glaucoma medication adherence in patients who viewed video-based educational tools.^[19]^ Yet, systematic reviews of the existing literature surrounding interventions to improve glaucoma medication adherence indicate there is insufficient evidence to support the promulgation of any existing glaucoma medication adherence tool.^[20]^

We developed the CNN utilized in the present study to address the clinical need for an effective patient-centered tool capable of improving medication adherence rates and subsequent visual outcomes in patients with glaucoma. Patients with irreversible vision loss report relying on other senses, such as hearing, to process the environment around them.^[21]^ Because our CNN converts real-time visual input into audio-based output, it provides a useful method for patients with glaucoma to remain adherent to their medications and preserve visual function. Widespread implementation of our CNN could provide opportunities for glaucoma specialists to foster patient-forward dialogues and healthy patient–physician partnerships that emphasize patient autonomy and self-determination, values that have been demonstrated to improve quality of life in patients with chronic disease.^[22]^

Our study is a single-center study with a narrowly-defined participant sample, which may limit the generalizability of our findings. Moreover, the methodology underlying the order in which medications were presented to patients in our identification activity may have introduced an element of systematic bias; using a fixed-order sequence of medications for both non-CNN and CNN cohorts may reflect the ease of identifying the medications included in each cohort rather than the effect of the CNN. Additional studies that assess the utility of our CNN need to be performed in a randomized manner to reduce the risk of bias and determine the efficacy of this tool in different clinical contexts. Further training of our CNN could incorporate medications used to treat other ophthalmic pathologies, such as retinal or corneal disease. Expanding the heterogeneity of our dataset may improve its scope and expand access to a valuable technology, which would promote better patient outcomes across all ophthalmology practices.

Overall, we found that our CNN smartphone application independently improves medication identification accuracy and time, especially in patients with glaucoma. This tool was also self-rated by study participants as improving the ease of eye drop medication identification. Our findings suggest the implementation of this CNN may be an effective intervention to improve medication compliance and prevent vision loss in patients with glaucoma.

##  Financial Support and Sponsorship 

The research reported in this manuscript was supported by the Institute for Clinical and Translational Science of the National Institutes of Health under award number T35DK128788. KYL also received funding from the Mentoring for Advancement of Physician Scientists (MAPS) as well as the Clinician Scientist Award grants from the American Glaucoma Society. The authors also acknowledge a Research to Prevent Blindness unrestricted departmental grant. The content in this manuscript is solely the responsibility of the authors and does not represent the official views of the National Institutes of Health, the American Glaucoma Society, or Research to Prevent Blindness.

##  Conflicts of Interest

KYL is a consultant for Bausch and Lomb and Johnson and Johnson. He also receives funding support from Zeiss for work unrelated to the present study.

Supplementary Table 1, Supplementary Table 2, Supplementary Table 3

**Supplementary Table 1:** Text processing algorithm results

**Medication**	**Image count**	**Accurate count**	**Accuracy**	**No match count**
Alphagan	102	96.0	0.941	6.0
Combigan	92	69.0	0.750	23.0
Cosopt	90	58.0	0.644	32.0
Dorzolamide (orange cap)	102	88.0	0.863	14.0
Latanoprost	100	61.0	0.610	39.0
Pred Forte	102	95.0	0.931	7.0
Pred Forte (off-brand)	101	95.0	0.941	6.0
Rhopressa	103	59.0	0.573	44.0
Rocklatan	94	51.0	0.543	43.0
Vigamox	101	75.0	0.743	26.0
Vigamox (off-brand)	101	79.0	0.782	22.0
**Total (excluding no matches)**	**826**	**826**	**1.000**	**0**
**Total (including no matches)**	**1088**	**826**	**0.759**	**262**

**Supplementary Table 2:** Participant survey questions

**Without smartphone application**	**With smartphone application**
“On a scale of 1–5 (with 1 being very easy and 5 being very difficult), how easy was it to identify the eye drop bottles with your naked eye?	“On a scale of 1–5 (with 1 being very easy and 5 being very difficult), how easy was it to identify the eye drop bottles using the CNN smartphone application?”
“Which features of the medication bottle are you using to identify the correct medication (e.g., text, color, shape)?”	

**Supplementary Table 3:** Participant-reported medication bottle features used for identification by the naked eye

**Bottle feature**	**Count**
Bottle text	53 (77.94%)
Bottle color	15 (22.06%)
Cap color	14 (20.59%)
Bottle shape/size	4 (5.88%)
Bottle font	1 (1.47%)
